# Draft genome sequence data of *Bacillus subtilis* strain *9407*, isolated from healthy apples in China

**DOI:** 10.1016/j.dib.2020.105143

**Published:** 2020-02-08

**Authors:** Qingchao Zeng, Jianbo Xie, Yan Li, Xiaofei Gu, Qi Wang

**Affiliations:** a College of Plant Protection, Key Laboratory of Plant Pathology, Ministry of Agriculture, China Agricultural University, Beijing, China; b College of Biological Sciences and Technology, Key Laboratory of Genetics and Breeding in Forest Trees and Ornamental Plants, Ministry of Education, Beijing Forestry University, Beijing, China

**Keywords:** *Bacillus*, Draft genome, Biocontrol and illumina

## Abstract

*Bacillus subtilis* strain 9407 is an endophyte which was isolated from healthy apples from an infested orchard that exhibits strong inhibitory activity against *Botryosphaeria dothidea*. Whole-genome sequencing of *B. subtilis* 9407 was performed using the Illumina Hiseq platforms. Here, we report the draft genome sequence of *B. subtilis* strain 9407 containing 16 scaffolds (4,062,615 bp), 4033 coding sequences, and an average 43.66% G + C content. The genome contains genes responsible for the production of several bioactive secondary metabolites, including the lipopeptides fengycin and surfactin. The genome information will provide fundamental knowledge of the organism. This whole-genome shotgun data has been deposited at DDBJ/EMBL/GenBank under the accession numbers PISO00000000.

**Table:** Specifications Table

Subject	Biology
Specific subject area	Microbiology and genomics
Type of data	Draft genome sequence data in FASTA format, figure and image
How data were acquired	Genome sequencing with Illumina Hiseq at Beijing Berrygenomics Bioinformatics Technology Co., Ltd.
Data format	Analyzed and assembled genome sequences
Parameters for data collection	Genomic DNA was extracted from a pure culture of B. subtilis 9407
Description of data collection	Whole-genome sequencing, assembly, and annotation
Data source location	B. subtilis 9407 was isolated from healthy apple in China
Data accessibility	This whole-genome shotgun data has been deposited at DDBJ/EMBL/GenBank under the accession numbers PISO00000000 (https://www.ncbi.nlm.nih.gov/nuccore/PISO00000000). The sequence data have been registered in the NCBI SRA database under the accession number SSR8935609 (https://www.ncbi.nlm.nih.gov/sra/?term&equals;SRR8935609) and SRR8935610 (https://www.ncbi.nlm.nih.gov/sra/?term&equals;SRR8935610).

**Table:** 

**Value of the Data** • The draft genome sequence of *B. subtilis* 9407 provides fundamental knowledge of this organism and insight for biotechnological application in agriculture. • The draft genome sequence data are useful for comparative genomic analysis of *Bacillus* species. • It will accelerate functional genomics research.

## 1 Data

Plant pathogens are one of the major challenges for sustainable food production and ecosystem stability worldwide [1]. Producers have been relying heavily on agrochemicals because they are a reliable and economical method to protect crops. However, the heavy use of chemicals has negatively affected the environment. Biocontrol formulations, as an alternative to chemical pesticides, have shown good application prospects in sustainable agriculture [2,3]. *Bacillus* spp. are biocontrol agents that have been extensively studied owing to their strong stress resistance and outstanding environmental adaptability. Many *Bacillus* strains are known to produce a diverse spectrum of secondary metabolites with antimicrobial activity [4,5]. *Bacillus subtilis* is a model Gram-positive bacterium that has been widely used for plant disease control for several decades [6]. *B. subtilis* is widespread in nature. The species neither causes disease in humans or animals nor pollutes the environment; in addition, it can produce a variety of antibiotics and inhibitory enzymes, such as iturin, bacilysin, and fengycin, with broad-spectrum antimicrobial/antifungal activity [6].

*B. subtilis* 9407 was isolated from healthy apples from an infested orchard in China. Our previous result showed that strain 9407 exhibits strong antagonistic activity toward apple ring rot and 18 other pathogens that cause plant diseases in apples, grapes, pears and other plants [7]. Here, we report the draft genome sequence of strain 9407 to facilitate further studies on pathways related to biocontrol and broaden the current understanding of the biological control mechanism of *B. subtilis* 9407. The draft genome of the strain 9407 was obtained by direct sequencing of genomic DNA using Illumina sequencing technology. After quality filtering, we obtained 6,963,036 (1.3 Gb for short insert) and 8,288,121 (1.54 Gb for long insert) paired-end reads.

The draft genome of *B. subtilis* 9407 contains 16 scaffolds with a total length of 4,062,615 bp with an *N*_*50*_ of 2,111,374 bp. The genome contains 4033 protein-coding genes (CDSs), 8 rRNA genes, and 62 tRNAs genes, with a G + C content of 43.65%. The protein-coding genes have an average length of 884 bp and account for 89.21% of the genome sequence. Some of these predicted genes and functions, such as those related to chemotaxis-related proteins, antimicrobial biosynthesis, biofilm formation, are known to be associated with growth promotion and biocontrol. The average nucleotide identity value between strain 9407 and 168^T^ was obtained by the JSpecies [8] software to exceed 98%.

Subsequent analyses of the genome content of *B. subtilis* 9407 and its comparison with phylogenetically related strains will help to determine key aspects of its interaction with the plants.

## 2 Experimental design, materials, and methods

*B. subtilis* 9407 was cultivated as previously described [7]. A single colony of *B. subtilis* 9407 was cultured in Lurina-Bertani medium at 37 °C overnight. Then, the bacterial cells were centrifuged and pelleted for DNA extraction. The genomic DNA of *B. subtilis* 9407 was extracted using the phenol-chloroform method [9] and sequenced at Beijing Berrygenomics Bioinformatics Technology Co., Ltd. Genomic DNA was used for construction of 350 bp and 2 kb libraries, using the TruSeq Nano DNA Sample Preparation Kit and Mate Pair Library Prep Kit v2, respectively. The manufacturer's instructions were followed in both cases. The libraries were sequenced on Illumina Hiseq 2500 in 2 × 100 paired-end format which achieved approximately 741x coverage. Adapters and low-quality sequences were removed using the software of Cutadapt and Sickle, respectively [10,11]. Assembly of filtered Illumina reads was done using the software SOAP*denovo* version 2.04 which the kmer value was 83 [12]. Gaps inside scaffolds were closed by using GapCloser version 1.12 [12]. Annotation was performed using PROKKA software [13].
